# Evaluation of global *Cenchrus* germplasm for key nutritional and silage quality traits

**DOI:** 10.3389/fnut.2022.1094763

**Published:** 2023-02-02

**Authors:** Sultan Singh, Tejveer Singh, Krishan Kunwar Singh, Manoj Kumar Srivastava, Madan Mohan Das, Sanat Kumar Mahanta, Neeraj Kumar, Rohit Katiyar, Probir Kumar Ghosh, Asim Kumar Misra

**Affiliations:** ^1^Plant Animal Relationship Division, ICAR-Indian Grassland and Fodder Research Institute, Jhansi, India; ^2^Crop Improvement Division, ICAR-Indian Grassland and Fodder Research Institute, Jhansi, India

**Keywords:** *Cenchrus* germplasm, ensiling, energy value, methane, sugar

## Abstract

*Cenchrus* is important genera of grasses inhabiting tropical pastures and the Indian grasslands system. Its forage value is well established to sustain nomadic livestock and wildlife. This study deals with the evaluation of the representative set of global *Cenchrus* germplasm collection with 79 accessions belonging to six species (*C. ciliaris, C. setigerus, C. echinatus, C. myosuroides, C. pennisetiformis, and C. biflorus)* at flowering stage. Crude protein (CP), neutral detergent fiber (NDF), acid detergent fiber (ADF), cellulose, and lignin values were in the range of 61.1–136, 640–749, 373–490, 277–375, and 35.6–75.50 g kg^−1^DM, respectively, while sugar contents varied from 11.6 to 101 mg g^−1^ DM. From the evaluated germplasm, 14 accessions of *C. ciliaris* having >70 mg g^−1^ DM sugar contents were selected and further evaluated for protein, fiber, carbohydrate and protein fractions, palatability indices, *in vitro* CH_4_ production, and ensiling traits. Protein contents were lower in EC397323 (61.8) and higher in IG96-96 (91.5), while the NDF, ADF, cellulose, and lignin contents varied between 678–783, 446–528, 331–405, and 39.6–62.0 g kg^−1^DM, respectively. The carbohydrate and protein fractions of selected accessions differed (*p* < 0.05), and the sugar contents varied (*p* < 0.05) between 74.6 and 89.6 mg^−1^g DM. Dry matter intake (DMI) and relative feed value (RFV) of accessions varied (*p* < 0.05) and were in the range of 1.53–1.77% and 58.2–73.8 g kg^−1^ DM, respectively. The total digestible nutrients (TDNs), digestible energy (DE), and metabolizable energy (ME) of selected accessions varied between 362–487 g kg^−1^ DM, 6.62–8.90, and 5.42–7.29 Mj kg^−1^ DM, respectively. *In vitro* gas and CH_4_ production (24 h) varied (*p* < 0.05) between 73.1 to 146 and 7.72 to 21.5 ml/g, respectively, while the degraded dry matter (g kg^−1^ DM) and CH_4_ (ml/g DDM) ranged between 399–579 and 17.4–47.2, respectively. The DM contents at ensiling, silage pH, and lactic acid contents of accessions differed (*p* < 0.05) and ranged between 185–345 g kg^−1^ DM, 5.10–6.05, and 1.39–23.3 g kg^−1^ DM, respectively. Wide genetic diversity existed in germplasm and selected *C. ciliaris* accessions for protein fiber, energy, sugar, and other nutritional traits. Silage prepared from EC397366, IG96-96, IG96-50, and EC397323 had pH and lactic acid contents acceptable for moderate to good quality silage of tropical range grasses.

## 1. Introduction

Grasses constitute up to 48% of all biomass fed to livestock globally (1), and the habitat of natural pastures, rangelands, forests, community lands, etc. serves as one of the major roughage sources for ruminants across the globe and usually constitutes more than 60% of the diet for small ruminants. Tropical grasses are nutritionally poor than temperate grasses, and other cereal forage crops [oat, maize, sorghum, barley, etc., Minson (2)] and their yield and nutritive value vary with species, growth stage, season, soil nitrogen status, and fertilizer application. Grasses are usually fed as green and hay and hardly conserved as silage primarily due to low dry matter, less water-soluble carbohydrate (WSC) contents, higher buffering capacity, and low energy contents (3, 4), which restrict the fermentation process and the subsequent adoption of tropical grass silage technology (5). Success in ensiling of grasses is governed by their readily available carbohydrate, and it is sufficiently high to promote the lactic acid bacteria to produce lactic acid to reduce pH during fermentation for subsequent preservation (6). According to Haigh (7), fresh grass should contain a minimum of 37.0 g kg^−1^ water-soluble carbohydrates or about 150 g kg^−1^ WSC on a dry weight basis to prepare good quality silage without silage additives. In addition, fodder species and their developmental stage are also important pre-ensiling factors responsible for silage quality (8).

The *Cenchrus* genus of the grass family has many species, which can tolerate a wide range of soil types and moisture conditions found globally including Asia, Africa, Australia, and the United States of America (9). In India, *Cenchrus* is an important component of *Dichanthium-Cenchrus-Lasiurus*-type grassland cover with coverage of >436,000 km^2^ (10). *Cenchrus species* mainly *Cenchrus ciliaris* (Buffel grass) and *Cenchrus setigerus* are important pasture grasses in the tropics (11), which are commonly used as a forage grass in India (12). It is drought-tolerant and a well-fertilized *C. ciliaris* crop may yield up to 24 t DM ha^−1^ (13) with a yield range of 2–18 t DM ha^−1^ without fertilizer. At the early flowering stage, hay prepared is of medium quality and rarely made into silage due to lower sugar contents and usually low moisture contents in the semi-arid regions. Efforts have been made to breed its cultivars for improved nutritive value and higher fodder yield, particularly in Australia. In India, a global collection of *Cenchrus* species are maintained at ICAR-Indian Grassland and Fodder Research Institute, and also, few varieties have been developed for higher biomass (14). However, no research efforts have been put to evolve the varieties for silage making (ensiling properties) but the need for Indian tropical grasslands due to the climatic situation, which favor surplus availability of fodder during monsoon months (mid-September–mid-November) and growth dormancy afterward. Keeping this in view, a multidisciplinary project was initiated on the evaluation of *Cenchrus* germplasm for higher sugar contents (>70 mg g^−1^ DM) required to initiate fermentation. So, in the present study, a 79 *Cenchrus* spp. genotypes were evaluated for yield, protein, and cell wall contents including sugar contents, while the sugar-rich (>70 mg g^−1^ DM) selected genotypes were evaluated for various nutritional parameters and ensiling properties.

## 2. Materials and methods

### 2.1. Location, germplasm maintenance, and multiplication of sugar-rich accessions

The study was carried out at ICAR-Indian Grassland and Fodder Research Institute, Central Research Farm, Jhansi, India (25°31′ N, 78°32′ E; 237 masl). The experimental site has a prevalence of semi-arid climatic conditions with extreme winter (as low as 2°C) and summer (43–46°C) temperatures. The edaphic/soil parameter consisted of deep, moderately well drained, and brown to dark grayish brown with a fine loamy texture. The optimum dose of fertilizers such as nitrogen (80 kg N ha^−1^), phosphorus (60 kg P/ha), and farmyard manure (30 t ha^−1^) was applied at sowing. Seeds of 79 accessions of *Cenchrus* spp. (*Cenchrus ciliaris* 53*, Cenchrus setigerus* 20*, Cenchrus echinatus* 3*, Cenchrus myosuroides* 1, *Cenchrus pennisetiformis* 1, *and Cenchrus biflorus* 1; Supplementary Table 1) representing corsets developed from over 600 global germplasm of *Cenchrus* spp. maintained in the Institute Gene Bank (14). When seedlings that reached the height of around 30 cm were transplanted with three checks (cv. IGFRI727, IGFRI3108, and IG-96-83) in an augmented randomized complete block design (15) during the rainy season. Each accession was transplanted in 1 × 3 m plots with 2 rows of plants/plot. Line-to-line and plant-to-plant distances were maintained at 50 × 50 cm, with a 1-m distance between two plots. Out of the 79, 14 accessions of *C. ciliaris* with >70 mg g^−1^ DM sugar contents were transplanted in RCBD during the rainy season in three replications. Each accession was planted in 4 × 3 m plots with six rows of plants/plots. Line-to-line and plant-to-plant distances were maintained at 50 × 50 cm with 1-m spacing between two plots.

### 2.2. Sample collection, processing, and drying

Samples of each accession from *Cenchrus* germplasm and selected sugar-rich accessions of *C. ciliaris* were harvested at the flowering stage in the rainy season from each row for nutritional evaluation. Immediately after harvesting, fresh forage yield was recorded using a digital portable balance. For the dry matter (DM) and dry matter yield (DMY), estimation samples were dried at 100°C for 72 h and at 60°C for 72 h for chemical/biochemical estimations (16). Dried samples were stored in the plastic sample containers (Tarson make) after fine grounding through a 1-mm sieve using a Willey mill for further nutritional and *in vitro* analyses.

### 2.3. Ensiling of sugar-rich accessions

Samples of IG99-124, IG97-379, IG-97-377, IG97-403, EC397323, IG96-87, EC400605, IG97-378, EC397366, EC397379, CC-14-1, IG96-96, IG96-89, and IG96-50 accessions were harvested in the forenoon (September 2016) and wilted for 2 h. Samples were chaffed (1–1.5 cm) through a manually operated chaffing machine and filled in the plastic containers (25.5 cm long × 13 cm diameter wide 5 kg volume) in triplicate for each accession. The chaffed samples filled were pressed manually with a hand and broad-based wooden rod to exclude as much air as possible, and then, the containers were capped and sealed with adhesive tape for ensiling. After 45 days of ensiling silage, containers were opened and representative samples were analyzed for silage DM, pH, lactic acid, and chemical composition.

### 2.4. Chemical analyses

#### 2.4.1. Chemical composition

Dry matter (930.15), N (976.05), ether extract (EE, 920.39), and ash (932.05) contents of *Cenchrus* genotypes and selected sugar-rich accessions were determined as per the standard protocol of AOAC (17). The obtained nitrogen values were multiplied by 6.25 to get CP-values. Samples of neutral detergent fiber (NDF), acid detergent fiber (ADF), cellulose, and lignin (sa) were estimated sequentially (18) using the fiber analyzer (Fibra Plus FES 6, Pelican, Chennai, India). Both NDF and ADF were expressed inclusive of residual ash. Heat stable α-amylase and sodium sulfite were not used in NDF determination. Lignin (sa) was determined by the solubilization of cellulose with 72% sulfuric acid in the ADF residue (18). Cellulose was calculated as the difference between ADF and lignin (sa) in the sequential analysis. Hemicellulose was calculated as the difference between NDF and ADF.

#### 2.4.2. Sugar contents

Total sugar contents of germplasm and selected accessions were estimated by the Anthrone method using glucose as standard (19). For this, 100 mg of ground sample (1-mm sieve) was treated with 10 ml of 80% ethanol in the water bath (80°C) for 30 min. The contents were centrifuged at 10,000 rpm for 10 min, the supernatant was collected in a volumetric flask, and the volume was made up to 25 ml. From this, 1 ml was further diluted to 50 ml, from this, 0.25 ml was taken in a tube, and 2.25 ml of distilled water and 5 ml of 0.2% Anthrone reagent were added. The mixture was boiled for 7 min and cooled, and the blue color developed was measured using a UV spectrophotometer (LABINDIA3000) at 630 nm.

#### 2.4.3. Carbohydrate fractions

Cornell Net Carbohydrate and Protein (CNCP) system (20) was used to determine the carbohydrate fractions of sugar-rich *C. ciliaris* accessions. This system further divides the carbohydrate components into four fractions based on their degradation rate; C_A:_ rapidly degradable sugars; C_B1:_ intermediately degradable starch and pectin; C_B2:_ slowly degradable cell wall; and C_C:_ unavailable/lignin bound cell wall.

Total carbohydrate (tCHO g kg^−1^ DM) was determined by subtracting CP, EE, and ash contents from 1,000. The difference between NDF and neutral detergent-insoluble protein (NDIP) was used to calculate the structural carbohydrates (SCs), and the difference between tCHO and SC (21) was estimated to calculate the non-structural carbohydrate (NSC). Starch was determined by extracting grass samples in 80% ethyl alcohol to solubilize free sugars, lipids, pigments, and waxes. The residue rich in starch was solubilized with perchloric acid and the extract was treated with anthrone-sulfuric acid to determine glucose calorimetrically using the glucose standard (19).

#### 2.4.4. Protein fractions

The CP fractions of sugar-rich accessions were partitioned into five fractions according to the Cornell Net Carbohydrate and Protein System [CNCPS; (20)] as modified previously (22). These are fraction P_A_ and non-protein N, which are calculated as the difference between total N and true CP N precipitated with sodium tungstate (0.30 M) and 0.5 M sulfuric acid; fraction P_B1_, buffer-soluble protein, determined as the difference between true protein and buffer-insoluble protein, estimated with borate-phosphate buffer (pH 6.7–6.8) and freshly prepared 0.10 sodium azide solution. Fraction P_B2_, neutral detergent-soluble protein, was estimated as the difference in buffer-insoluble protein and ND-insoluble protein, whereas fraction P_B3_, acid detergent-soluble CP, was estimated as the difference between ND-insoluble protein and acid detergent-insoluble CP. Fraction P_C_ is assumed to be indigestible.

Neutral detergent-insoluble protein (NDIP), acid detergent-insoluble protein (ADIP), and non-protein nitrogen (NPN) were determined following the standard method (22). For NDIP and ADIP, samples extracted with neutral detergent and acid detergent solutions, respectively, were analyzed as Kjeldahl N × 6.25 using a semi-auto analyzer (Kel Plus Classic-DX Pelican India). For NPN estimation, samples were treated with sodium tungstate (0.30 M) and filtered, and residual nitrogen was determined by the Kjeldahl procedure. Non-protein nitrogen of the sample was calculated by subtracting residual nitrogen from total nitrogen. Soluble protein (SP) was estimated by treating the samples in borate-phosphate buffer, pH 6.7–6.8, consisting of monosodium phosphate (Na_2_PO_4_.H_2_O) 12.2 g L^−1^, sodium tetra borate (Na_2_B_4_O_7_.10H_2_O) 8.91 g L^−1^, and tertiary butyl alcohol 100 mL L^−1^and freshly prepared 10% sodium azide solution (23). The N estimated in the residue gives the insoluble protein fraction. The SP was calculated by subtracting the insoluble protein from the total CP.

#### 2.4.5. Gross energy estimation and calculations for DDM, DMI, RFV, and energy

*Cenchrus* germplasm and selected sugar-rich accessions, dry matter intake (DMI), digestible dry matter (DDM), relative feed value (RFV), total digestible nutrients (TDN), and net energy for different animal functions, i.e., lactation (NE_L_), gain (NE_G_), and maintenance (NE_M_), were calculated using the equations [DMI = 120/NDF; DDM = 88.9–0.779^*^ADF; RFV = (DDM^*^DMI)^*^0.775; TDN = 104.97–(1.302^*^ADF); NE_L_ =(TDN^*^0.0245)−0.012; NE_G_ = (TDN^*^0.029)−1.01; NE_M_ = (TDN^*^0.029)−0.29] of Undersander et al. (24). Digestible energy (DE, KJ g^−1^ DM; DE = TDN^*^0.04409) and metabolizable energy (ME, KJ g^−1^ DM) values were calculated using the equations of Fonnesbeck et al. (25) and Khalil et al. (26), respectively. Metabolizable energy was calculated as DE × 0.821.

### 2.5. *In vitro* incubation

#### 2.5.1. Donor animals and inoculum preparation

Overall, four adult male *Jalauni* sheep with a mean body weight of 38.7 ± 0.473 kg were used as inoculum donors. These animals were maintained on a sole berseem hay diet and had free access to clean drinking water. Rumen liquor was collected in a pre-warmed thermos from each animal before feeding using a perforated tube from the stomach with the help of a vacuum pressure pump. Rumen liquor collected from each animal was filtered through four layers of muslin cloth and mixed well to have the composite sample, kept at 39°C in a water bath, and gassed with CO_2_ till used for mixing with incubating buffer media.

*In vitro* gas production was estimated as per the pressure transducer technique (27). The incubation medium was formulated by sequential mixing of buffer solution (NH_4_HCO_3_ and NaHCO_3_), macro-mineral solution, micro-mineral solution, and resazurin solution (28). Samples (1.0 g) of air-dry *Cenchrus* sugar-rich accessions were weighed into three serum bottles (150 ml of capacity). In total, three serum bottles without substrate were used as blank cultures. Sample and control serum bottles were gassed briefly with CO_2_ before adding 65 ml of medium. Bottles were continuously fluxed with CO_2_, and then, 3 ml of reducing solution was added to each bottle. The gassing of bottles with CO_2_ continued till the pink color turned colorless. Before inoculation, the gas pressure transducer was used to adjust the head-space gas pressure in each bottle (to adjust the zero reading on the LED display). Serum bottles were inoculated with 8 ml of ruminal fluid inoculum using a 10-ml syringe. Inoculated bottles were sealed and incubated at 39°C. Samples were incubated in triplicates and gas production (ml) was measured at 24 h of incubation. The whole process was repeated on a different day.

#### 2.5.2. Methane measurements

At 24 h of incubation, methane in total gas was measured from three bottles incubated for each of the *Cenchrus* accession by gas chromatography (Nucon 5765 Microprocessor controlled gas chromatograph, Okhla, New Delhi, India) equipped with a stainless-steel column packed with Porapak-Q and a Flame Ionization Detector. Gas (1 ml) was sampled from gas produced using a Hamilton syringe and injected manually (pull and push methods of sample injection) into a gas chromatograph calibrated with standard methane and CO_2_. Methane was also measured from three serum bottles used as blanks for the correction of methane produced from the rumen inoculum. Methane measured was related to total gas to estimate its concentration (29). Short-chain fatty acids (SCFA) were calculated using 24 h gas production as described by Getachew et al. (30). Microbial mass (MBM) and partitioning factor (PF) were calculated as described by Blümmel et al. (31).

### 2.6. Silage analysis

For DM estimation, 100 g of the fresh silage sample was dried in a hot air oven at 60°C till the constant weight is achieved and then corrected for DM using the equation of Kaiser and Kerr (32) as estimated true DM (%) = 4.686 + (0.89 × oven DM %). For silage pH and lactic acid estimation, a 20 g of fresh silage sample was put in a beaker, and to this, 100 ml of tepid water was added. Beaker was kept in a water bath shaker (30°C) for 30 min, and contents were agitated manually and filtered through filter paper. The filtrate was mixed well, a portion of it was used to measure pH using a digital pH meter (Systronic 360), and the remaining filtrate was used for lactic acid estimation as described by Barker and Summerson (33). For this, 1 ml of extract was added to the tubes and 0.05 ml of 4% CuSO4 and 6 ml of concentrated H_2_SO_4_ were added drop by drop with continuous shaking. Tubes were kept in a boiling water bath for 5 min and cooled at room temperature, and then, 0.1 ml of P-hydroxyphenyl reagent was added drop by drop and incubated in a shaker water bath at 30°C for 30 min. The blue color developed was measured at 560 nm using UV-spectrophotometer (LABINDIA3000).

### 2.7. Statistical analysis

To describe the variability among the accessions, univariate statistics including means and ranges were used, which were obtained for each trait based on the accessions. Data on dry matter yield were subjected to statistical analysis using the descriptive statistics on adjusted means estimated by the R package for augmented design (34). Data were subjected to a one-way analysis of variance of SPSS 17.0 to test the differences between *Cenchrus* accessions for chemical composition, sugar contents, carbohydrate and protein fractions, energy values, digestibility and *in vitro* gas and methane production, and silage quality (pH, lactic acid, and DM contents). Variable means were compared for significance (*p* < 0.05) level using Duncan's multiple-range test (35). Euclidean distance as a measure of dissimilarity and incremental sums of squares as a grouping strategy was utilized for clustering the accessions based on their morphological traits using the “cluster” package of SAS statistical software (36). Dendrograms were constructed based on the fusion level to examine the similarities in the pattern of performance among the accessions.

## 3. Results

### 3.1. Biomass and nutritional variability in *Cenchrus* spp. germplasm

**Figure 1:**
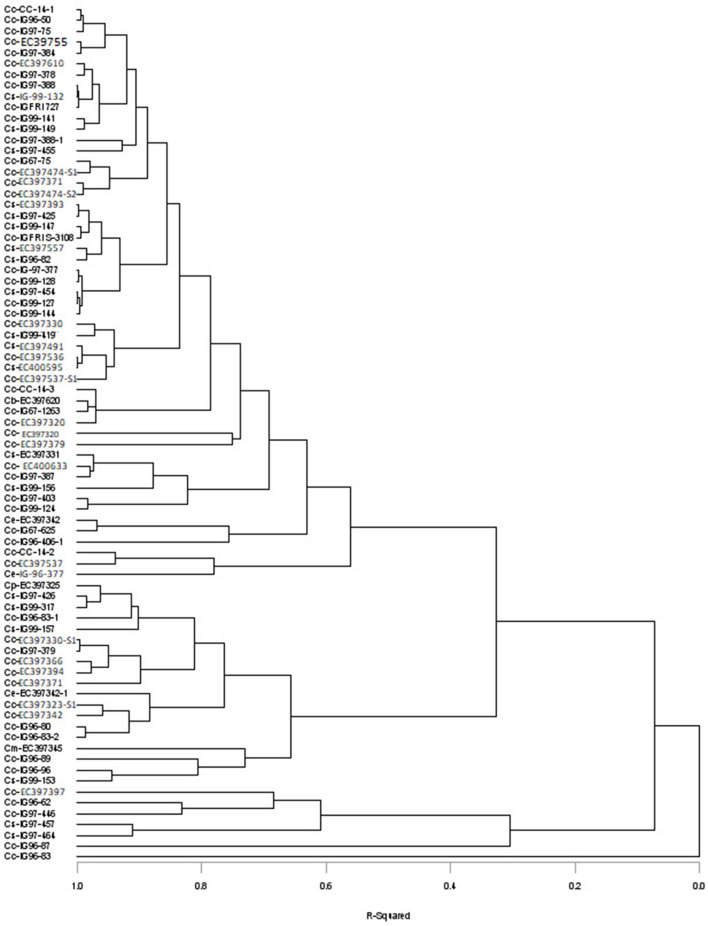
Distribution of different *Cenchrus* species accessions into different clusters based on nutritional and biochemical traits (Cc, *Cenchrus ciliaris*; Cs, *Cenchrus setigerus*; Ce, *Cenchrus echinatus*; Cm, *Cenchrus myosuroides*; Cp, *Cenchrus pennisetiformis*; Cb, *Cenchrus biflorus*).

The variance and range showed that sufficient variability exits in germplasm evaluated for DMY, chemical composition (CP, NDF, ADF, cellulose, and lignin), sugar contents, and other nutritional traits (Table 1), and the values for these traits for individual accession are given in Supplementary Table 1. The DMY of evaluated *Cenchrus* species germplasm varied from 1.85 to 34.27 t/ha; CP, NDF, ADF, cellulose, and lignin contents varied between 61.1–136, 640–750, 370–510, 250–400, and 31.0–97.0 g kg^−1^ DM, with their mean values of 88.0, 694, 426, 321, and 53.2 g kg^−1^ DM, respectively. Soluble sugar contents of *Cenchrus* germplasm varied widely in the range of 11–101mg/g with a mean value of 57.07 mg/g. Mean values of total digestible nutrients (TDNs), digestible energy (DE), metabolizable energy (ME), net energy for lactation, net energy for maintenance, and net energy for the gain of the *Cenchrus* germplasm were 495 g kg^−1^ DM, 9.02, 7.41, 4.51, 5.42, and 1.82 Mj/Kg, respectively. The mean values of dry matter intake (DMI), digestible dry matter (DDM), and relative feed values (RFVs) for the *Cenchrus* germplasm were 1.73%, 553 g kg^−1^ DM, and 72.75%, respectively. The cluster analysis placed 79 accessions into five clusters (R^2^ = 0.4). Clusters one and two included single accession each, cluster three included five accessions of *C. ciliaris* and *C. setigerus*, cluster four contain 19 accessions of *C. ciliaris, C. setigerus, C. echinatus, C. myosuroides*, and *C. pennisetiformis*, and cluster five included 53 accessions of *C. ciliaris, C. setigerus, C. echinatus*, and *C. biflorus* (Figure 1).

**Table 1:** Mean performance of *Cenchrus* species for chemical components and nutritional quality traits.

**Species^†^**	***C. ciliaris***			***C. setigerus***			***C. echinatus***			***C. biflorus***	***C. pennisetiformis***	***C. myosuroides***
**Traits^!^**	**Mean ±SE**	**Variance**	**Range**	**Mean ±SE**	**Variance**	**Range**	**Mean ±SE**	**Variance**	**Range**	**Mean**	**Mean**	**Mean**
DMY	12.28 ± 0.97	50.02	4.02–34.27	7.38 ± 0.68	9.15	2.48–12.27	4.14 ± 0.97	2.81	3.15–6.08	7.80	4.20	1.85
CP	88.5 ± 2.0	2.20	61.1–136	86.3 ± 1.5	0.48	75.0–103	93.1 ± 10.1	3.08	73.2–106	105	80.3	75.0
NDF	695 ± 2.9	4.43	640–749	692 ± 4.6	4.27	652–728	712 ± 15.1	6.85	689–740	689	671	665
ADF	427 ± 2.9	4.44	373–490	426 ± 4.2	3.49	390–474	424 ± 19.3	11.22	386–451	446	411	388
Cellulose	326 ± 2.7	3.97	277–375	309 ± 2.8	1.58	285–334	305 ± 9.2	2.54	287–319	341	309	304
Lignin	51.8 ± 0.9	0.46	35.6–70.7	56.5 ± 1.6	0.49	45.6–75.5	61.1 ± 4.7	0.68	53.6–69.9	55.8	52.2	37.8
Sugar	60.36 ± 2.92	452	11.65–101.47	53.84 ± 2.48	123	33.59–72.84	27.74 ± 8.58	221	10.69–37.92	58.3	56.9	62.0
DMI	1.73 ± 0.01	0.00	1.60–1.85	1.74 ± 0.01	0.00	1.65–1.84	1.69 ± 0.04	0.00	1.62–1.74	1.74	1.79	1.8
DDM	552 ± 2.2	2.65	502–595	552 ± 3.3	2.18	515–581	554 ± 15.2	6.96	533–584	537	564	583
RFV	72.57 ± 0.47	11.67	64.21–81.04	72.77 ± 0.79	12.49	67.25–82.16	70.71 ± 3.46	35.97	64.55–76.53	69.58	76.7	80.79
TDN	493 ± 3.7	7.23	411–564	494 ± 5.4	5.91	432–542	498 ± 25.2	19.04	462–547	469	514	544
DE	9.05 ± 0.07	0.24	7.55–10.34	9.07 ± 0.10	0.20	7.93–9.94	9.13 ± 0.46	0.64	8.48–10.02	8.6	9.43	9.99
ME	7.43 ± 0.06	0.16	6.20–8.49	7.45 ± 0.08	0.13	6.51–8.16	7.50 ± 0.38	0.43	6.96–8.23	7.06	7.74	8.2
NE_L_	4.53 ± 0.04	0.07	3.70–5.25	4.54 ± 0.06	0.06	3.91–5.02	4.57 ± 0.26	0.20	4.21–5.07	4.28	4.74	5.05
NE_M_	5.41 ± 0.04	0.11	4.43–6.27	5.43 ± 0.07	0.09	4.68–6.00	5.47 ± 0.31	0.28	5.04–6.06	5.12	5.66	6.03
NE_G_	1.80 ± 0.05	0.13	0.58–2.67	1.84 ± 0.06	0.07	1.18–2.35	1.91 ± 0.31	0.29	1.52–2.52	1.62	2.07	2.38

^†^*Cenchrus ciliaris, Cenchrus setigerus, Cenchrus echinatus, Cenchrus myosuroides, Cenchrus pennisetiformis, and Cenchrus biflorus*.

^!^DMY, Dry matter yield t/ha; CP, Crude protein g kg^−1^ DM; NDF, Neutral detergent fibre g kg^−1^ DM; ADF, Acid detergent fibre g kg^−1^ DM; Cellulose, g kg^−1^ DM; Lignin, g kg^−1^ DM; Sugar = mg g^−1^ DM; DMI, Dry matter intake%; DDM, Digestible dry mater g kg^−1^ DM; RFV, Relative feed value %; TDN, Total digestible nutrients g kg^−1^ DM; DE, Digestible energy Mj kg^−1^ DM; ME, Metabolizable energy Mj kg^−1^ DM; NE_*L*_, Net energy for lactation Mj kg^−1^ DM; NE_*G*_, Net energy for growth/gain Mj kg^−1^ DM; NE_*M*_, Net energy for maintenance Mj kg^−1^ DM.

### 3.2. Sugar contents and chemical composition of *C. ciliaris* accessions

Sugar contents of 14 sugar-rich *C. ciliaris* accessions, *viz*. IG99-124, IG97-379, IG-97-377, IG97-403, EC397323, IG96-87, EC400605, IG97-378, EC397366, EC397379, IG96-96, IG96-89, IG96-50, and CC-14-1 were more than 70 mg g^−1^ DM required for ensiling (Table 2). Sugar contents of accessions differed (*p* < 0.05) and varied between 74.6 (IG99-127) and 89.6 mg g^−1^ DM (EC397323). Accessions of CP, NDF, ADF, cellulose, and lignin contents varied (*p* < 0.05), and their mean values were 76.4, 737, 478, 351, and 49.1 g kg^−1^ DM, respectively (Table 2). The OM and EE contents of sugar-rich accessions varied (*p* < 0.05) with mean values of 856 and 20.5 g kg^−1^ DM, respectively.

**Table 2:** Sugar contents (mg g^−1^DM) and chemical composition (g kg^−1^ DM) of sugar-rich *Cenchrus ciliaris* genotypes.

**Accessions**	**Sugar**	**OM**	**CP**	**EE**	**NDF**	**ADF**	**Cellulose**	**Lignin**	**Hemi cellulose**
IG99-124	80.1^abc^	849^de^	68.7^bc^	20.2^cd^	735^def^	432^a^	326^a^	39.8^a^	303^h^
IG97-379	78.6^a^	865^g^	77.8^de^	21.3^de^	722^bc^	507^ef^	343^cdef^	60.0^d^	215^ab^
IG-97-377	76.0^a^	881^i^	72.4^cd^	20.9^cde^	738^ef^	4724^c^	348^efg^	39.6^a^	266^f^
IG97-403	74.6^a^	875^h^	73.5^cd^	16.4^a^	777^h^	516^f^	355^g^	51.7^c^	260^def^
EC397323	79.1^ab^	866^g^	61.8^a^	17.3^ab^	763^g^	455^b^	339^bcde^	46.8^abc^	308^h^
IG96-87	88.9^bc^	841^b^	75.9^de^	21.1^cde^	717^b^	471^c^	342^cdef^	42.1^ab^	245^cd^
EC400605	81.3^abc^	851^de^	83.9^f^	22.7^e^	733^cde^	469^c^	353^fg^	62.0^d^	264^ef^
IG97-378	81.3^abc^	853^e^	77.5^de^	21.0^cde^	746^f^	488^d^	346^defg^	50.9^c^	258^def^
EC397366	81.9^abc^	847^cd^	76.8^de^	22.0^de^	735^def^	528^g^	395^i^	48.8^bc^	207^a^
EC397379	78.8^ab^	882^i^	65.2^ab^	21.6^de^	783^h^	505^e^	405^j^	48.5^bc^	281^g^
IG96-96	82.5^abc^	840^b^	91.5^g^	18.9^bc^	678^a^	451^b^	334^abcd^	59.6^d^	227^b^
IG96-89	82.5^abc^	860^f^	85.5^f^	22.3^de^	740^ef^	446^b^	331^abc^	51.3^c^	294^gh^
IG96-50	89.6^c^	842^bc^	82.1^ef^	20.7^cde^	726^bcd^	483^d^	372^h^	46.2^abc^	243^c^
CC-14-1	81.9^abc^	832^a^	76.6^de^	21.2^de^	724^bcd^	473^c^	328^ab^	40.4^ab^	250^cde^
Mean	81.2^abc^	856	76.4	20.5	737	478	351	49.1	258
SEM	0.85	2.44	1.27	0.32	4.08	4.28	3.71	1.27	4.75
*P*-value	0.075	< 0.0001	< 0.0001	< 0.0001	< 0.0001	< 0.0001	< 0.0001	< 0.0001	< 0.0001

CP, Crude protein; OM, Organic matter; EE, Ether extract; NDF, Neutral detergent fiber; ADF, Acid detergent fiber. Superscripts a–h within columns differ significantly between rows at (*P* < 0.05).

### 3.3. Protein and carbohydrate fractions of *C. ciliaris* accessions

Total carbohydrate (tCHO), non-structural carbohydrates (NSCs), and structural carbohydrates (SCs) of *C. ciliaris* accessions differed (*p* < 0.05) from 730 to 795, 27.1 to 67.9, and 662 to 765 g kg^−1^ DM, respectively (Table 3). Similarly, the carbohydrate fractions, namely, C_A_ C_B1_, C_B2_, and C_c_ varied (*p* < 0.05) across the accessions, and their mean values were 46.5, 56.1, 742, and 155 g kg^−1^ tCHO, respectively. *C. ciliaris* accessions protein fractions, *viz*. P_A_, P_B1_, P_B2_, P_B3_, and P_C_ differed (*p* < 0.05) and ranged between 163–425, 172–492, 85.8–206, 44.3–194, and 81.6–152 g kg^−1^ CP, respectively (Table 4).

**Table 3:** Carbohydrate fractions of sugar-rich *Cenchrus ciliaris* genotypes.

**Accessions**	**tCHO**	**NSC**	**SC**	**C_A_**	**C_B1_**	**C_B2_**	**C_C_**
IG99-124	760^g^	46.8^d^	713^cd^	68.8^cd^	32.5^a^	773^c^	126^ab^
IG97-379	766^h^	61.0^e^	706^bc^	86.8^d^	50.0^b^	675^b^	188^ef^
IG-97-377	788^i^	64.6^e^	723^de^	68.3^cd^	46.1^b^	765^c^	120^a^
IG97-403	785^i^	27.1^a^	758^g^	29.1^ab^	47.2^b^	766^c^	158^cd^
EC397323	787^i^	42.2^bcd^	745^f^	46.9^bc^	45.2^ab^	765^c^	143^abcd^
IG96-87	742^cd^	43.2^de^	699^b^	45.3^b^	60.0^bcd^	759^c^	136^abcd^
EC400605	744^cd^	24.7^a^	719^de^	33.6^ab^	67.4^de^	699^b^	200^f^
IG97-378	755^fg^	30.5^abc^	725^e^	17.7^a^	75.0^e^	745^c^	162^de^
EC397366	748^de^	29.4^ab^	719^de^	15.3^a^	76.7^e^	751^c^	156^cd^
EC397379	795^j^	30.3^abc^	765^g^	17.8^a^	58.0^bcd^	778^c^	146^abcd^
IG96-96	730^a^	67.9^e^	662^a^	110^e^	55.4^bcd^	638^a^	196^f^
IG96-89	752^ef^	27.8^a^	724^de^	25.8^ab^	65.1^cde^	745^c^	1647^de^
IG96-50	739^bc^	33.8^abc^	705^bc^	42.6^b^	54.8^bcd^	752^c^	150^bcd^
CC-14-1	735^ab^	33.0^abc^	702^b^	43.1^b^	52.0^bc^	773^c^	132^abc^
Mean	759	40.2	719	46.5	56.1	742	155
SEM	3.32	2.38	3.99	4.53	2.07	6.60	4.20
*P*-value	< 0.0001	< 0.0001	0.051	< 0.0001	< 0.0001	< 0.0001	< 0.0001

tCHO, Total carbohydrates g kg^−1^ DM; NSC, Non-structural carbohydrates g kg^−1^ DM; SC, Structural carbohydrates g kg^−1^ DM; C_A_, Rapidly degradable sugars g kg^−1^ tCHO; C_B1_, Intermediately degradable starch and pectins g kg^−1^ tCHO; C_B2_, Slowly degradable cell wall g kg^−1^ tCHO; C_C_, Unavailable/lignin-bound cell wall g kg^−1^ tCHO. Superscripts a–j within columns differ significantly between rows at (*P* < 0.05).

**Table 4:** Protein fractions (g kg^−1^ CP) of sugar-rich *Cenchrus ciliaris* accessions.

**Genotype**	**P_A_**	**P_B1_**	**P_B2_**	**P_B3_**	**P_C_**
IG99-124	234^abc^	362^bcde^	86.5^a^	222	95.4^ab^
IG97-379	193^ab^	450^efg^	139^cde^	92.7^bc^	124^cde^
IG-97-377	290^bc^	289^b^	206^f^	108^bcd^	107^bc^
IG97-403	236^abc^	387^cdef^	137^bcde^	143^de^	97.8^ab^
EC397323	314^c^	312^bc^	85.8^a^	136^cde^	152^f^
IG96-87	254	396^def^	120^abcd^	96.4^bcd^	132^def^
EC400605	227^abc^	455^fg^	165^ef^	44.3^a^	109^bc^
IG97-378	215^abc^	408^defg^	100^abcd^	171^ef^	106^bc^
EC397366	306^bc^	325^bcd^	157^de^	77.0^ab^	134^def^
EC397379	204^abc^	414^defg^	96.0^abc^	140^cde^	146^ef^
IG96-96	238^abc^	410^defg^	178^ef^	92.8^bc^	81.6^a^
IG96-89	283^bc^	414^defg^	115^abcd^	71.0^ab^	11.71^bcd^
IG96-50	163^a^	492^g^	101^abcd^	144^de^	99.9^ab^
CC-14-1	425^d^	17.2^a^	93.0^ab^	194^fg^	116^bcd^
Mean	256	377	127	124	115
SEM	12.4	13.7	6.42	8.18	3.41
*P*-value	0.002	< 0.0001		< 0.0001	< 0.0001

P_A_, Non-protein nitrogen; P_B1_, Buffer-soluble protein; P_B2_, Neutral detergent-soluble protein; P_B3_, Acid detergent-soluble protein; P_C_, Indigestible protein. Superscripts a–g within columns differ significantly between rows at (*P* < 0.05).

### 3.4. Energy contents of *C. ciliaris* accessions

Energy contents of sugar-rich *C. ciliaris* accessions in terms of TDN, DE, and ME differed (*p* < 0.05), and their mean values were 427 g kg^−1^ DM, 7.78 Mj kg^−1^ DM, and 6.42 Mj kg^−1^ DM, respectively (Table 5). Accessions net energy efficiency for different animal functions, *viz*. maintenance (NE_M_), lactation (NE_L_), and growth (NE_G)_ varied (*p* < 0.05) from 3.81 to 5.30, 3.19 to 4.43, and 0.16 to 1.66 Mj kg^−1^ DM, respectively.

**Table 5:** Energy contents of sugar-rich *Cenchrus ciliaris* accessions.

**Accessions**	**TDN**	**DE**	**ME**	**NE_L_**	**NE_M_**	**NE_G_**
IG99-124	487^g^	8.90^g^	7.29^h^	4.43^h^	5.30^g^	1.66^g^
IG97-379	389^bc^	7.12^c^	5.84^bc^	3.44^bc^	4.14^bc^	0.50^c^
IG-97-377	434^e^	7.95^e^	6.50^ef^	3.89^ef^	4.68^e^	1.04^e^
IG97-403	377^b^	6.87^b^	5.67^b^	3.31^b^	3.97^b^	0.33^b^
EC397323	457^f^	8.32^f^	6.83^g^	4.14^g^	4.97^f^	1.32^f^
IG96-87	436^e^	7.95^e^	6.54^f^	3.93^f^	4.72^e^	1.08^e^
EC400605	439^e^	8.03^e^	6.58^f^	3.97^f^	4.72^e^	1.08^e^
IG97-378	415^d^	7.58^d^	6.21^d^	3.73^d^	4.43^d^	0.79^d^
EC397366	362^a^	6.62^a^	5.42^a^	3.19^a^	3.81^a^	0.17^a^
EC397379	395^c^	7.20^c^	5.92^c^	3.52^c^	4.22^c^	0.58^c^
IG96-96	463^f^	8.45^f^	6.91^g^	4.18^g^	5.01^f^	1.37^f^
IG96-89	469^f^	8.57^f^	7.04^g^	4.26^g^	5.09^f^	1.45^f^
IG96-50	420^d^	7.66^d^	6.29^de^	3.77^de^	4.51^d^	0.87^d^
CC-14-1	433^e^	7.91^e^	6.50^ef^	3.89^ef^	4.68^e^	1.04^e^
Mean	427	7.78	6.42	3.85	4.60	0.95
SEM	5.56	0.025	0.020	0.014	0.030	0.016
*P*-value	< 0.0001	< 0.0001	< 0.0001	< 0.0001	< 0.0001	< 0.0001

TDN, Total digestible nutrients g kg^−1^ DM; DE, Digestible energy Mj Kg^−1^ DM; ME, Metabolizable energy Mj Kg^−1^ DM; NE_L_, Net energy for lactation Mj Kg^−1^ DM; NE_G_, Net energy for growth/gain Mj Kg^−1^ DM; NE_M_, Net energy for maintenance Mj Kg^−1^ DM. Superscripts a–h within columns differ significantly between rows at (*P* < 0.05).

### 3.5. Palatability attributes of *Cenchrus* accessions

The DMI, DDM, and RFV for sugar-rich accessions also differed (*p* < 0.05) and their mean values were 1.63%, 516 g kg^−1^ DM, and 65.28%, respectively (Table 6).

**Table 6:** Palatability attributes of sugar-rich *Cenchrus ciliaris* accessions.

**Accessions**	**DMI**	**DDM**	**RFV**
IG99-124	1.63^cde^	552^g^	69.9^h^
IG97-379	1.66^fg^	494^bc^	63.6^c^
IG-97-377	1.62^cd^	521^e^	65.6^de^
IG97-403	1.54^a^	486^b^	58.2^a^
EC397323	1.57^b^	534^f^	65.1^d^
IG96-87	1.67^g^	522^e^	67.7^fg^
EC400605	1.64^efg^	524^e^	66.4^def^
IG97-378	1.61^c^	509^d^	63.4^c^
EC397366	1.63^cde^	477^a^	60.4^b^
EC397379	1.53^a^	497^c^	59.0^a^
IG96-96	1.77^h^	538^f^	73.8^i^
IG96-89	1.62^cd^	541^f^	68.0^g^
IG96-50	1.65^efg^	512^d^	65.7^de^
CC-14-1	1.66^fg^	520^e^	66.8^efg^
Mean	1.63	516	65.3
SEM	0.006	3.32	0.641
*P*-values	< 0.0001	< 0.0001	< 0.0001

DMI, Dry matter intake %; DDM, Digestible dry mater g kg^−1^ DM; RFV, Relative feed value%. Superscripts a–i within columns differ significantly between rows at (*P* < 0.05).

### 3.6. Gas and methane production from sugar-rich *Cenchrus* accessions

*In vitro* gas and methane production from *C. ciliaris* accessions differed (*p* < 0.05) with the mean values of 108 ml g^−1^ DM and 14.8 ml g^−1^ DM, respectively (Table 7). Methane production ml g^−1^ DDM and DDM (g kg^−1^ DM) varied (*P* < 0.05) between 17.4–47.2 and 399–579, respectively, across the accessions. Gas fermentation parameters, namely, partition factor (PF), short-chain fatty acid (SCFA), microbial mass (MBM), and efficiency for microbial mass production (EMBM) varied (*p* < 0.05) across the accessions. The values of PF and EMBM were highest and SCFA were lowest for accession G96-50.

**Table 7:** *In vitro* gas and methane production from sugar-rich *Cenchrus ciliaris* accessions.

**Genotypes**	**Gas** **ml g^−1^**	**CH_4_** **ml g^−1^**	**DDM** **g kg^−1^ DM**	**CH_4_ ml g^−1^ DDM**	**PF mg** **DDM ml^−1^**	**SCFA** **mmol g^−1^ DM**	**MBM** **mg g^−1^ DM**	**EMBM** **mg mg^−1^**
IG96-87	97.6^abcd^	7.72^a^	439^ab^	17.4^a^	4.50^abcd^	2.16^abcd^	224^bc^	0.51^bcd^
EC400605	111^bcd^	11.2^abc^	501^bcde^	22.8^ab^	4.59^bcd^	2.46^bcd^	256^c^	0.51^bcd^
IG97-378	104^bcd^	10.6^ab^	458^abcd^	23.1^ab^	4.40^abcd^	2.32^bcd^	228^bc^	0.49^bc^
EC397366	104^bcd^	15.3^bcd^	531^ef^	28.5^abc^	5.16^de^	2.30^bcd^	303^cde^	0.57^cde^
EC397379	122^def^	17.3^cde^	509^cde^	33.9^bcd^	4.18^abcd^	2.72^def^	239^bc^	0.47^bc^
IG96-96	101^bcd^	14.3^bcd^	530^ef^	26.6^abc^	5.37^de^	2.23^bcd^	309^cde^	0.58^cde^
IG96-89	104^bcd^	15.3^bcd^	482^bcde^	31.4^bc^	4.69^cd^	2.31^bcd^	253^c^	0.52^bcd^
IG96-50	73.1^a^	13.6^abcd^	517^def^	26.2^abc^	7.07^f^	1.62^a^	356^de^	0.69^e^
CC-14-1	116^cde^	19.8^de^	524^def^	37.7^cde^	4.67^cd^	2.57^cde^	269^cd^	0.51^bcd^
IG99-124	110^bcd^	15.3^bcd^	399^a^	38.5^cde^	3.64^abc^	2.44^bcd^	156^ab^	0.39^ab^
IG97-379	141^ef^	19.1^de^	447^abc^	43.5^de^	3.30^ab^	3.13^ef^	136^a^	0.30^a^
IG-97-377	146^f^	21.5^e^	457^abcd^	47.2^e^	3.24^a^	3.23^f^	137^a^	0.30^a^
EC397323	87.4^ab^	12.7^abc^	544^ef^	23.4^ab^	6.34^ef^	1.94^ab^	352^de^	0.65^de^
IG97-403	93.8^abc^	14.3^bcd^	579^f^	24.8^ab^	6.39^ef^	2.08^abc^	373^e^	0.64^de^
Mean	108	14.9	493	30.5	4.79	2.40	254	0.51
SEM	3.21	0.66	7.97	1.44	0.176	0.07	11.9	0.02
*P*-value	< 0.0001	< 0.0001	< 0.0001	< 0.0001	< 0.0001	< 0.0001	< 0.0001	< 0.0001

DDM, Degraded dry matter; PF, Partition factor mg of *in vitro* degraded dry matter to ml of gas thereby produced; SCFA, Short-chain fatty acid; MBM, Microbial mass; EMBP, Efficiency of microbial protein production. Superscripts a–f within columns differ significantly between rows at (*P* < 0.05).

### 3.7. Silage composition

Silage pH and lactic acid contents for evaluated *C. ciliaris* accessions differed (*p* < 0.05) and ranged between 5.11 (EC397366) to 6.07 (EC397379) and 3.71 (IG97-403) to 23.7 g kg-^1^ DM (EC397366), respectively (Table 8).

**Table 8:** *Cenchrus ciliaris* accessions silage composition.

**Accessions**	**Silage composition**†			
	**pH**	**Lactic acid g kg^−1^ (Fresh)**	**Lactic acid g kg^−1^ DM)**	**DM at ensiling g kg^−1^ DM**
IG96-87	5.54^bcd^	5.63^ef^	21.6^ef^	185^a^
EC400605	5.68^cde^	1.08^a^	5.01^ab^	212^ab^
IG97-378	5.77^def^	2.98^abcde^	5.90^abc^	228^abc^
EC397366	5.11^a^	7.15^f^	23.7^f^	305^de^
EC397379	6.07^g^	3.15^abcde^	9.22^bcd^	343^e^
IG96-96	5.48^bc^	4.30^bcdef^	17.8^e^	239^abc^
IG96-89	5.66^cde^	4.45^cdef^	19.3^ef^	232^abc^
IG96-50	5.46^bc^	2.85^abcde^	11.1^cd^	254^bcd^
CC-14-1	5.95^fg^	2.15^abc^	8.71^bcd^	246^bc^
IG99-124	5.77^def^	2.63^abcde^	12.5^d^	213^ab^
IG97-379	5.70^cde^	3.83^abcde^	7.70^abcd^	274^cd^
IG-97-377	5.54^bcd^	5.30^def^	6.91^abc^	258^bcd^
EC397323	5.41^b^	2.50^abcd^	14.3^de^	233^abc^
IG97-403	5.48^bc^	1.28^ab^	3.71^a^	245^bc^
IG3158	5.77^def^	1.80^abc^	7.90^abcd^	225^abc^
Mean	5.60	3.40	11.6	246
SEM	0.034	0.30	0.89	6.16
*P*-value	< 0.0001	< 0.0001	< 0.0001	< 0.0001

^†^LA, Lactic acid; DM, Dry matter. Superscripts a–g within columns differ significantly between rows at (*P* < 0.05).

## 4. Discussion

### 4.1. Chemical composition

The chemical composition of feed/fodder is one of the important determinants of its nutritive value. *C. ciliaris* germplasm and sugar rich accessions had CP more than 70.0g kg^−1^ DM required for sustained rumen microbial activity (37). CP content is a measure of nutritional quality (38), our germplasm and sugar rich accessions had CP similar to 80.0 g kg^−1^ DM which is considered adequate for the maintenance of beef cattle (39).

Information on the nutritive value of *C. echinatus, C myosuroides*, and *C. pennisetiformis* is not available; however, the CP, NDF, ADF, cellulose, and lignin along with energy values have been reported for *C. biflorus* and *C. setigerus* (40, 41), and our values are more or less within the range of their reported values. The OM, CP, NDF, ADF, cellulose, and lignin of five new genotypes of *CC* differed (*p* < 0.05) and were in the range of 881–904, 80–96, 687–738, 485–519, 367–432, and 34–60 g kg^−1^ DM, and the mean values of 893, 87, 713, 492, 400, and 43 g kg^−1^ DM (42) were more or less similar to the values recorded for the accessions evaluated in the present study. In the study, mean values of OM, CP, NDF, cellulose, and lignin contents of 78 new genotypes of *CC* evaluated in Mexico (43) were 861, 82, 734, 413, and 31 g kg^−1^ DM, respectively. Melesse et al. (44) reported that C ciliaris grass at pre flowering growth had CP, EE, NDF, ADF, cellulose, and lignin contents of 82, 14.5, 601, 373, 342, and 26.7 g kg^−1^ DM, respectively. Ashraf et al. (45) reported higher EE (27.0–53.0) and protein contents (132–175 g kg^−1^ DM) of 10 *C. ciliaris* accessions from the Cholistan desert of Pakistan than our EE and CP contents. Saini et al. (46) evaluated six cultivars/species of *CC* for 2 years (2003–2004) and found that CP contents ranged from 94.1 to 157 g kg^−1^ DM in 2003 and 37.2 to 101 g kg^−1^ DM in 2004 during 1st and 2nd cut. Cenchrus ciliaris from light and heavy grazed rangeland (Gemeda and Hassan (47) had mean value of 873, 41.0, 19.5, 682, 418, 360, and 55.0 g kg^−1^ DM for OM, CP, EE, NDF, ADF, cellulose, and lignin, respectively, lies within the range of our values except for CP, which was lower than our values. Bezabih et al. (48) reported that *C. ciliaris* collected from six transects of grazing areas of semi-arid savanna grassland in the Mid Rift Valley of Ethiopia had mean values of 889, 563, and 96.0 g kg?1 DM for OM, NDF and CP, respectively. Coelho et al. (49) reported that the mean values of CP, NDF, ADF, and lignin were 98.0, 685, 337, and 26.0 and 111, 688, 349, and 37.0 g kg^−1^ DM for *C. ciliaris* harvested four times at 60-90 days growth under stockpiled and grazing conditions. CP and ash contents of 11 ecotypes of *C. ciliaris* grass grown in the semi-arid lands of Kenya ranged between 66.4–109 and 112–152 g kg^−1^1 DM, respectively (50). Jonathan et al. (51) reported that C. ciliaris grass hay fed to sheep had CP, NDF, ADF, OM, and EE of 46.0, 725, 542, 898, and 86.5 g kg^−1^1 DM, respectively.

### 4.2. Sugar contents

The sugar content is a measure of rapidly fermentable energy available from a forage/feed and plays an important role in ruminant nutrition as required for both efficient rumen microbial fermentation and lactic acid production during the ensiling process. Sugar contents are of significant importance for ensiling (52) as it is the main source of nutrients for microbes to produce lactic acid and a sugar content level of 80 mg g^−1^ DM, which is desirable. *C. ciliaris* germplasm showed (*p* < 0.05) that the differences in sugar contents were lowest in IG67-625 (11.0 mg g^−1^ DM) and highest in EC397323 (101 mg g^−1^). The *CC* germplasm mean soluble sugar contents (57.1 mg g^−1^ DM) were lower than the desired level while sugar-rich accession mean sugar contents were more than 70 mg/g DM required for ensiling. Low levels of sugar and water-soluble carbohydrates in tropical grasses limit the fermentative capacity and result in low-quality silage (53). Aminah et al. (54) reported that the sugar content (WSC) of six types of tropical grass ranged between 12.6 and 98.8 mg g^−1^ DM, which are in agreement with the evaluated germplasm and selected accession sugar contents. Water-soluble carbohydrate contents of *Kikuyu grass, Seteria, Rhodes, Signal, Napier, Guinea, and Paspalum grass* were 45, 48, 30, 86, 99, 30, and 31 mg g^−1^ DM, respectively (55).

### 4.3. Carbohydrate and protein fractions

The mean tCHO of seven types of tropical grass at 56 days of cutting age varied from 730 to 836 g kg^−1^ DM (56), and our values of sugar-rich accessions (730–795 g kg^−1^ DM) are within this range. Brandstetter et al. (57) reported that carbohydrate fractions C_A+B1_, C_B2_, and C_C_ of Jiggs Bermuda grass in different seasons (fall, winter, spring, and summer) varied between 240–376, 539–650, and 84.9–128 g kg^−1^ tCHO, respectively. Sa et al. (58) reported that carbohydrate fractions C_A+B1_, C_B2_, and Cc of *Cynodon dactylon, Brachiaria brizantha*, and *Panicum maximum* grasses at 28, 35, and 54 days of cutting age ranged between 165–255, 346–449, and 110–263 g kg^−1^ tCHO. These workers further reported that tCHO and NFC contents of *Cynodon dactylon, Brachiaria brizantha*, and *Panicum maximum* grasses ranged between 728–827 and 20–90 g kg^−1^ DM, respectively. The NSCs of 15 types of tropical grass from 87 to 223 g kg^−1^ DM (59) were higher than our values (24.7–67.9 NSC g kg^−1^ DM) and similar to 42.0 g kg^−1^ DM recorded by Jonathan et al. (51) for *C. ciliaris*. Higher C_C_ and lower C_B2_ for IG97-379, EC400605, and IG96-96 accessions may be attributed to their higher lignin and lower NDF contents as the forages with high NDF have a higher proportion of C_B2_ fraction, and the increase in the fraction C_C_ can be partly attributed to the increased lignin concentration in NDF (60).

*C. ciliaris* high sugar accessions P_C_ fraction varied (*p* < 0.05) with the mean value of 115 g kg^−1^ CP, which is in the agreement with the range of 100–150 g kg^−1^ CP as reported earlier (61, 62). Fraction C is the insoluble N in acid detergent solution (ADIN) and is associated with lignin, tannin–protein complexes, and Maillard products. This fraction represents the unavailable protein and is assumed to have zero ruminal and intestinal digestibility. In our study, P_C_ fraction varied (*p* < 0.05) between 81.6 and 152 g kg^−1^ CP is within the range of 90–180 g kg^−1^ CP as reported by Sanderson and Wedin (63) and Hoekstra et al. (64). Jonathan et al. (51) reported that *C. ciliaris* P_A_, P_B1_, P_B2_, P_B3_, and P_C_ fractions of protein were 345, 152, 137, 195, and 480 g kg^−1^ CP, respectively, which were inconsistent with our protein fraction values except the mean value of P_B3_ (124 g kg^−1^ CP). Braga et al. (65) reported (*p* < 0.05) differences in protein fractions in grass species and their harvesting age. Protein fraction P_A_ composed of NPN has a higher rate of ruminal degradation, which was lower in *Andropogon* (120–130 g kg^−1^ CP) than in *C. ciliaris* and Massai (160–170 g kg^−1^ CP) at 63 days of cutting age. Grasses P_B2_, P_B3_, and P_C_ protein fractions ranged between 280–340, 270–310, and 213–273 g kg^−1^ CP, respectively, at 63 days of cutting age. Brandstetter et al. (57) reported that the protein fractions P_A_, P_B1_, P_B2_, P_B3_, and P_C_ of Jiggs Bermuda grass in different seasons (fall, winter, spring, and summer) between 407–550, 138–139, 100–157, 125–148, and 80–115 g kg^−1^ CP, respectively, and their P_B2_, P_B3_, and P_C_ values corroborate with our values of sugar-rich accessions for these fractions. Sa et al. (58) reported that protein fractions P_A_, P_B1+B2_, P_B3_, and P_C_ of *Cynodon dactylon, Brachiaria brizantha*, and *Pancum maximum* grasses at 28, 35, and 54 days of cutting age ranged between 164 to 287, 251 to 538, 116 to 349, and 91 to 182 g kg^−1^ CP, respectively, which are more or less similar to our observations.

### 4.4. Energy contents

The calculated mean ME values of *Cenchrus* germplasm (7.41 Mj kg^−1^ DM) and sugar-rich accessions (6.42 Mj kg^−1^ DM) were lower than those reported by Getachew et al. (66) for 17 grass samples (7.7–13.6 Mj kg^−1^ DM) and are inadequate to fulfill the energy requirements for the maintenance of growing cattle (8.8 Mj kg^−1^ DM; 37). Mlay et al. (67) reported that TDN, DE, and ME contents of 10 types of tropical grass ranged between 342–609 g kg-^1^ DM, 5.92–11.26, and 4.85–9.23 Mj kg^−1^ DM, respectively, and our values of *Cenchrus* germplasm (411–549 g kg-^1^ DM, 7.99–10.31, and 6.17–8.44 Mj kg^−1^ DM) and sugar-rich accessions (362–487 g kg-^1^ DM, 6.62–8.90, and 5.42-7.29 Mj kg^−1^ DM) lie within these values. The higher TDN and DE values of IG99-124 may be due to lower ADF and lignin contents (432 and 39.8 g kg-^1^ DM) as higher ADF and lignin contents reduce the nutrient utilization present in forages (68). Yigzaw (69) reported that the ME contents of *C. ciliaris* varied in the range of 7.65–9.02 Mj kg^−1^ DM during 60 to 120 days of crop growth. The effect of growth stage, season, and species on ME contents of tropical grasses has been reported earlier (51, 70). The net energy system recommends an animal energy requirement for different physiological functions, *viz*. tissue maintenance, tissue growth, and lactation (71). The NE_L_ contents of 4.38 and 4.85 Mj kg^−1^ DM for Napier and Pangola grasses, respectively, reported by Tikam et al. (70) are similar to the mean NE_L_ values of *Cenchrus* germplasm (4.51 Mj kg^−1^ DM) and higher than the mean NE_L_ contents of sugar-rich *C. ciliaris* accessions (3.85 Mj kg^−1^ DM). The NE_L_ values of IG99-124. EC397323, IG96-96, and IG96-98 accessions are almost similar to the values of Tikam et al. (70). *Cenchrus* germplasm and high sugar accessions had the adequate NE_M_ levels recommended for a mature beef cow [4.92–5.30 Mj kg^−1^ DM (71)].

### 4.5. Palatability attributes of *Cenchrus* accessions

Many indices to predict the forage quality for feeding ruminants have been developed (72) based on the chemical constituents of forages. Intake is one of the important indices to measure the nutrient availability of animals influenced by both diet and livestock species. Differences in DMI of *Cenchrus* germplasm (1.61–1.88%) and sugar-rich accessions (1.53–1.77%) may be attributed to the variation in their NDF contents. The mean NDF contents of both germplasm (694 g kg^−1^ DM) and sugar-rich accessions (737 g kg^−1^ DM) were beyond the range of 600–650 g kg^−1^ DM level, which is considered to influence the intake negatively (18). The DMI of tropical grasses at the flowering stage varied (*p* < 0.05) and ranged between 30.0 for *Panicum maximum* and 54.0g/kg w^075^ for *Brachiaria ruziziensis* and *Pennisetum purpureum* in sheep fed *ad lib* (73). Aguir et al. (74) reported DMI of 2.33% and 2.25% for Sudan grass and Elephant grass, respectively, in goats. Assoumaya et al. (75) reported that the voluntary intake of tropical forages is lower (1.95) than that of temperate forages (2.03%).

Forages with digestibility values of 500 g kg^−1^ DM or more can meet the energy requirements for the maintenance of grazing ruminants (76). The significant differences in DDM values of *C. ciliaris* germplasm (533–585 g kg^−1^ DM) and sugar-rich accessions (477–552 g kg^−1^ DM) may be attributed to the differences in their ADF and lignin contents as both nature and quantity of cell wall contents and cell contents of forages influence the DM degradability (37). Like our observations, Dessommes et al. (42) reported (*p* < 0.05) the differences in effective DM degradability of six *C. ciliaris* genotypes (550–663 g kg^−1^ DM). The *IVDMD* contents of 11 ecotypes of *C. ciliaris* ranged between 456 and 550 g kg^−1^ DM (50). Coelho et al. (49) reported *IVDMD* of 452 and 500 g kg^−1^ DM for *C. ciliaris* under stockpiled and grazing conditions.

Relative feed value (RFV) forage quality index combines the intake and digestibility into one unit (77). Alike our observations on RFV for *Cenchrus* germplasm (64.21–83.87%) and sugar-rich accessions (58.24–73.78%), Hackman et al. (78) reported a wide variability in RFV of 11 cool-season (71.5–130.0%) and four warm-season types of grass (88.0–165.0%), respectively. Suhaimi et al. (79) evaluated more than 900 samples of *Brachiaria decumbens* grass over 5 years (1999–2003) and observed that their RFV values ranged between 74.83 and 84.17%. Cinar and Hatipoglu (80) reported that the RFV of Dallis, Bermuda, and Rhodes grasses varied between 68.0–82.1, 75.0–93.7, and 72.8–86.7%, during 3 years of growth (2009–2011) and our RFV lies within these values.

### 4.6. Gas and methane production from sugar-rich *Cenchrus* accessions

*Cenchrus ciliaris* accessions differ (*p* < 0.05) in gas production with the mean value of 108 ml g^−1^ DM and are on the pattern of Ley de Coss et al. (81) who recorded (*p* < 0.05) the differences for *in vitro* gas production from four types of tropical grass (122–170 ml g^−1^ DM) at 24 h of incubation in bovine rumen liquor. Garcia and Dessommes (42) reported CH_4_ production of 4.38 ml g^−1^ DM of *C. ciliaris* at 24 h of fermentation while CH_4_ production for 16 types of grass ranged between 4.02 and 11.70 ml g^−1^ DM, which was lower than our CH_4_ values (7.72–19.80 ml g^−1^ DM). In contrast, Bezabiah et al. (48) reported higher gas and CH_4_ (202 and 40 ml g^−1^ DM) of *C. ciliaris* fermented for 24 h in rumen liquor of Holstein Friesian cattle. Similarly, Melesse et al. (44) recorded higher gas and methane production (204.5 and 34.5 ml g^−1^ DM) for *C. ciliaris* than our values. The authors further reported that the total gas and CH_4_ of 24 grass species ranged between 94 to 232 ml g^−1^ DM and 26 to 43 ml g^−1^ DM, respectively. The variation in methane production among *C. ciliaris* accessions may be partially attributed to their significant differences in chemical constituents such as CP, ash, ether extract, ADF, NDF, ADL, NDIN, ADIN, and NFC concentration. The ratio between methane to total gas production indicates that the methane emission potential per unit of OM degraded from forages, and in the present study, this ratio varied widely from 0.079 to 0.170 across the sugar-rich accessions, which shows the opportunity to select the accessions with low methane potential.

Accessions gas fermentation parameters, *viz*. partition factor (PF), short-chain fatty acid (SCFA), microbial mass (MBM), and efficiency for microbial mass production (EMBM) differed (*p* < 0.05). Accession IG96-50 higher values for PF and EMBM and lower for SCFA were consistent with the previous report, and the microbial mass and SCFA are inversely related (82, 83). Higher PF recorded for IG96-50 resulted in greater microbial mass as PF is the measure of the efficiency of microbial production. The amount of short-chain fatty acid produced is related to OMD and the energy content of the feed.

### 4.7. Silage quality

Typical concentrations of lactic acid in commonly fed silages range from 20.0 to 40.0 g kg^−1^ DM, but can be considerably higher in silages with low concentrations of DM (< 300 g kg^−1^ DM). The final pH of silage is affected by many factors but is most related to the concentration of lactic acid and buffering capacity of the crop. Silage prepared from *C. ciliaris* accessions had pH values (5.11–6.07) above the 3.8 to 4.2 ideal ranges (52) usually observed in corn or sorghum or oat silages. Silage pH values of EC397366 (5.11), IG96-96 (5.46), IG96-50 (5.46), and EC397323 (5.41) accessions are more or less in the acceptable range of tropical grasses with lactic acid contents of 23.7, 17.8, 11.1, and 14.3 g kg^−1^ DM, respectively. Harrison et al. (84) recommended that good grass silage should have pH < 4.47 and lactic acid between 40.0 and 70.0 g kg^−1^ DM, respectively. The pH values recorded for sugar-rich *Cenchrus* accessions are acceptable and consistent with the values reported for grasses (85, 86). Aminah et al. (54) reported that silages from *Seteria splendid* and *Pennisetum purpureum* had lower pH (4.07 and 3.96) and more lactic acid (24.7 and 25.3 g kg^−1^ DM) than other evaluated tropical grasses (4.71–5.32) and 10.4–18.4 g kg^−1^ DM) partially agree with our values of IG96-96, EC397366, EC397323, and IG96-50 accessions. The DM content of evaluated accessions except EC397366 was below the range of 300 g kg^−1^ DM desirable for ensiling grasses. Pitt (87) suggested that grasses ensiled below 300 g kg^−1^ D should have >100 g kg^−1^ DM soluble sugar for adequate fermentation to achieve the desired pH. Accessions having higher pH might have failed to provide adequate substrate (sugar) to lactic acid bacteria to produce lactic acid. Pinho et al. (88) recorded the pH of Buffel grass silage between 4.6 and 5.4 at 30 days of fermentation harvested at different heights. Li et al. (89) reported the pH and LA contents of *Paspalum plicatulum* grass 5.2 and 11.0 g kg^−1^ DM and 5.2 and 18.0 g kg^−1^ DM during 30 days of fermentation at 28 and 40°C temperature and 5.2 and 17.0 g kg^−1^ DM and 5.1 and 20.0 g kg^−1^ DM during 60 days of ensiling at 28 and 40°C temperature, respectively. Yahaya et al. (90) showed that silage of tropical grass (*Pennisetum purpurum*) had higher pH and lower lactic acid (5.45 and 9.00 g kg^−1^ DM) than temperate rye grass silage (3.86 and 19.0 g kg^−1^ DM). In another study, Arroquy et al. (91) recorded lower pH (4.04–4.47) and higher lactic acid (39.1–76.5 g kg^−1^ DM) for six warm season types of grass than our pH and lactic acid values. However, Vendramini et al. (92) reported higher pH (6.5–8.6) and lower lactic acid (1.00–19.0 g kg^−1^ DM) for warm season grasses except for Limpo grass (26.0 g kg^−1^ DM) than our pH and lactic acid values.

## 5. Conclusions

The results revealed wide genetic variability in *Cenchrus* germplasm and sugar-rich accessions for dry matter yield, protein, fiber, energy, and soluble sugar contents. Sugar-rich accessions also differ (*p* < 0.05) for carbohydrate fractions, protein fractions, *in vitro* gas and methane production, and silage quality (pH and lactic acid). Silage prepared from EC397366, IG96-96, IG96-50, and EC397323 accessions had pH and lactic acid contents acceptable for tropical range grasses. Nutritional evaluation of silage prepared from selected accessions may be undertaken using *in vivo* studies. The present global subset having wide variability could be utilized for the identification of genomic regions associated with key forage nutritional traits for future breeding programs. Selected accessions need to be introduced in rangelands and pastures to enhance their yield and quality for sustainable livestock production.

## Data availability statement

The original contributions presented in the study are included in the article/Supplementary material, further inquiries can be directed to the corresponding authors.

## Ethics statement

The animal study was reviewed and approved by Institute Animal Ethics Committee.

## Author contributions

SS, TS, and SKM: conceptualized the study. SS, TS, SKM, MMD, KKS, RK, and AKM: methodology and laboratory work. PKG, AKM, SS, and TS: resources and supervision. SS, TS, and MKS: investigation. SS, TS, and NK: data analysis and writing. All authors contributed to the article and approved the submitted version.
