# Supplementary material for: Evaluation of global Cenchrus germplasm for key nutritional and silage quality traits
Source: Front Nutr. 2023 Feb 2;9:1094763. doi: 10.3389/fnut.2022.1094763 (PMC9932515; doi:10.3389/fnut.2022.1094763)
Supplement: Supplementary file 1 [file Table_1.doc]

TABLE 1 Forage quality and biomass yield of *Cenchrus* spp. genotypes

| **Accessions**  **identity** | ***Species*** | **!Traits** | | | | | | | | | | | | | | | |
| --- | --- | --- | --- | --- | --- | --- | --- | --- | --- | --- | --- | --- | --- | --- | --- | --- | --- |
|  |  | **CP** | **NDF** | **ADF** | **Cellu**  **lose** | **Lignin** | **Sugar** | **DMI** | **DDM** | **RFV** | **TDN** | **DE** | **ME** | **NEL** | **NEM** | **NEG** | **DMY** |
| CC-14-1 | *C. ciliaris* | 95.8 | 697.1 | 437 | 336.4 | 57.9 | 84.59 | 1.72 | 544.0 | 70.15 | 480.7 | 8.82 | 7.24 | 4.4 | 5.26 | 1.74 | 5.51 |
| CC-14-2 | *C. ciliaris* | 99.5 | 745.5 | 447.3 | 340.5 | 61.2 | 53.52 | 1.61 | 535.9 | 64.21 | 467.3 | 8.57 | 7.04 | 4.26 | 5.1 | 1.6 | 7.10 |
| CC-14-3 | *C. ciliaris* | 102.5 | 697.6 | 434.6 | 347.5 | 56.9 | 48.5 | 1.72 | 546.1 | 71.89 | 483.8 | 8.87 | 7.29 | 4.43 | 5.3 | 1.67 | 7.40 |
| IG-67-1263 | *C. ciliaris* | 113.3 | 696.3 | 440.4 | 339.8 | 59.1 | 52.28 | 1.72 | 541.1 | 67.36 | 476.4 | 8.74 | 7.17 | 4.36 | 5.21 | 1.87 | 6.33 |
| IG-67-625 | *C. ciliaris* | 94.5 | 702.1 | 427.4 | 312.1 | 60.6 | 11.65 | 1.71 | 551.5 | 70.22 | 493.2 | 9.05 | 7.43 | 4.53 | 5.41 | 1.92 | 8.10 |
| IG-67-75 | *C. ciliaris* | 87.1 | 686.9 | 432 | 323.6 | 56.3 | 20.21 | 1.75 | 548.0 | 71.89 | 487.2 | 8.94 | 7.34 | 4.47 | 5.34 | 1.8 | 8.30 |
| EC397320 | *C. ciliaris* | 106.6 | 677.9 | 440.9 | 329.5 | 56.2 | 38.02 | 1.77 | 540.9 | 71.3 | 475.7 | 8.73 | 7.16 | 4.35 | 5.2 | 1.71 | 15.76 |
| EC397323 | *C. ciliaris* | 61.1 | 692.7 | 439.1 | 350 | 49.7 | 101.47 | 1.73 | 542.4 | 70.9 | 478 | 8.77 | 7.2 | 4.37 | 5.23 | 1.66 | 20.65 |
| EC397323-S1 | *C. ciliaris* | 92.4 | 683.4 | 400 | 289.6 | 52.8 | 48.14 | 1.76 | 573.3 | 76.51 | 528.9 | 9.7 | 7.96 | 4.89 | 5.84 | 2.25 | 13.27 |
| EC397330 | *C. ciliaris* | 82 | 720.5 | 433.4 | 312.8 | 66.3 | 38 | 1.67 | 547.2 | 71.08 | 485.5 | 8.9 | 7.31 | 4.45 | 5.32 | 1.58 | 7.26 |
| EC397330-S1 | *C. ciliaris* | 78.6 | 704.1 | 400.7 | 296.9 | 39.6 | 66.01 | 1.7 | 572.8 | 74.72 | 528 | 9.68 | 7.95 | 4.88 | 5.83 | 2.2 | 5.10 |
| EC397366 | *C. ciliaris* | 80.3 | 689 | 411.6 | 320.6 | 43 | 76.84 | 1.74 | 564.1 | 74.64 | 513.8 | 9.42 | 7.74 | 4.74 | 5.66 | 2.07 | 9.75 |
| EC397371 | *C. ciliaris* | 90.5 | 696.4 | 426.8 | 339.4 | 45.9 | 41.82 | 1.72 | 552.1 | 71.53 | 494 | 9.06 | 7.44 | 4.54 | 5.42 | 1.88 | 19.62 |
| EC397371-S1 | *C. ciliaris* | 101.1 | 730.4 | 414.8 | 330 | 70.7 | 28.14 | 1.65 | 561.6 | 69.76 | 509.6 | 9.35 | 7.67 | 4.69 | 5.61 | 2.05 | 5.25 |
| EC397379 | *C. ciliaris* | 69.7 | 727.9 | 420.8 | 328.5 | 47.7 | 83.61 | 1.65 | 556.8 | 69.03 | 501.8 | 9.2 | 7.56 | 4.62 | 5.52 | 1.98 | 10.87 |
| EC397394 | *C. ciliaris* | 98 | 716.8 | 407.7 | 308.6 | 41.5 | 45.24 | 1.68 | 567.2 | 72.11 | 518.9 | 9.52 | 7.81 | 4.79 | 5.72 | 2.14 | 7.12 |
| EC397397 | *C. ciliaris* | 73.2 | 712.3 | 456.3 | 367.7 | 44.5 | 47.23 | 1.68 | 528.9 | 67.53 | 455.6 | 8.36 | 6.86 | 4.14 | 4.96 | 1.37 | 6.66 |
| IG-96-50 | *C. ciliaris* | 96 | 683.7 | 434.8 | 347.7 | 55.3 | 86.02 | 1.76 | 545.8 | 71.53 | 483.6 | 8.87 | 7.28 | 4.43 | 5.3 | 1.79 | 28.08 |
| EC397474 | *C. ciliaris* | 108.1 | 684 | 399.6 | 300.8 | 44.8 | 66.76 | 1.75 | 573.7 | 77.1 | 529.4 | 9.71 | 7.97 | 4.9 | 5.85 | 2.21 | 24.66 |
| EC397474-S1 | *C. ciliaris* | 87.2 | 700.8 | 436.2 | 328.3 | 57.3 | 36.7 | 1.71 | 544.9 | 72.79 | 481.7 | 8.84 | 7.25 | 4.41 | 5.28 | 1.53 | 30.15 |
| EC397474-S2 | *C. ciliaris* | 75.1 | 695.3 | 429.8 | 332 | 46.3 | 34.74 | 1.73 | 549.8 | 72.59 | 490.1 | 8.99 | 7.38 | 4.5 | 5.38 | 1.74 | 14.05 |
| EC397536 | *C. ciliaris* | 93.2 | 715.7 | 430.6 | 329.1 | 55.2 | 45.15 | 1.68 | 549.1 | 69.79 | 489 | 8.97 | 7.36 | 4.48 | 5.36 | 1.78 | 8.78 |
| EC397537 | *C. ciliaris* | 99.6 | 749.2 | 435.5 | 341 | 53.1 | 30.38 | 1.6 | 545.3 | 66.06 | 482.7 | 8.85 | 7.27 | 4.42 | 5.29 | 1.71 | 14.80 |
| EC397537-S1 | *C. ciliaris* | 76.7 | 738.3 | 430.7 | 329.4 | 49.8 | 32.68 | 1.63 | 549.2 | 68.41 | 489 | 8.97 | 7.36 | 4.48 | 5.36 | 1.72 | 9.40 |
| EC397558 | *C. ciliaris* | 89.1 | 680.1 | 432.3 | 327.8 | 49.7 | 56.76 | 1.76 | 547.8 | 73.56 | 486.8 | 8.93 | 7.33 | 4.46 | 5.34 | 1.73 | 15.60 |
| IG-96-62 | *C. ciliaris* | 69.7 | 698 | 470.2 | 356.9 | 62.2 | 48.56 | 1.72 | 518.0 | 68.58 | 437.5 | 8.02 | 6.59 | 3.96 | 4.74 | 1.06 | 8.78 |
| EC397610 | *C. ciliaris* | 69.7 | 702 | 433.8 | 342 | 53.1 | 71.31 | 1.71 | 546.7 | 72.18 | 484.9 | 8.89 | 7.3 | 4.44 | 5.31 | 1.62 | 8.91 |
| EC397672 | *C. ciliaris* | 90.6 | 686 | 405 | 312.2 | 50 | 68.52 | 1.75 | 569.4 | 76.28 | 522.4 | 9.58 | 7.87 | 4.83 | 5.77 | 2.13 | 12.13 |
| IG-96-80 | *C. ciliaris* | 118.8 | 678.5 | 401.3 | 317.1 | 46.8 | 28.02 | 1.77 | 572.2 | 75.64 | 527.2 | 9.67 | 7.94 | 4.87 | 5.82 | 2.32 | 34.27 |
| IG-96-83 | *C. ciliaris* | 136.2 | 670.2 | 373 | 287.3 | 35.6 | 61.46 | 1.79 | 594.6 | 81.04 | 564 | 10.34 | 8.49 | 5.25 | 6.27 | 2.67 | 18.04 |
| IG-96-83-1 | *C. ciliaris* | 76.8 | 694.1 | 407.5 | 277.3 | 56.3 | 72.57 | 1.73 | 567.4 | 74.84 | 519.1 | 9.52 | 7.82 | 4.79 | 5.73 | 2.11 | 4.02 |
| IG-96-83-2 | *C. ciliaris* | 106.5 | 684.6 | 406.9 | 303.7 | 46.8 | 28.96 | 1.75 | 567.7 | 73.65 | 520 | 9.54 | 7.83 | 4.8 | 5.74 | 2.29 | 8.43 |
| IG-96-87 | *C. ciliaris* | 76.8 | 662.6 | 490.1 | 375.4 | 39.6 | 85.37 | 1.81 | 502.5 | 72.5 | 411.5 | 7.55 | 6.2 | 3.7 | 4.43 | 0.58 | 6.36 |
| IG-96-89 | *C. ciliaris* | 92.5 | 671.6 | 404.5 | 325.1 | 55.1 | 97.92 | 1.79 | 569.7 | 77.05 | 523 | 9.59 | 7.88 | 4.83 | 5.77 | 2.2 | 30.28 |
| IG-96-96 | *C. ciliaris* | 97.8 | 639.9 | 384.6 | 313.5 | 54 | 89.19 | 1.85 | 575.5 | 80.76 | 532.5 | 9.77 | 8.02 | 4.93 | 5.89 | 2.3 | 18.27 |
| EC400633 | *C. ciliaris* | 110.2 | 669.6 | 423.4 | 318 | 48.8 | 69.14 | 1.79 | 554.9 | 75.92 | 498.5 | 9.14 | 7.51 | 4.58 | 5.48 | 1.85 | 14.47 |
| IG-97-377 | *C. ciliaris* | 76.9 | 688.2 | 426 | 319.9 | 52.7 | 86.48 | 1.74 | 552.9 | 74.53 | 495.1 | 9.08 | 7.45 | 4.55 | 5.44 | 1.74 | 11.88 |
| IG-97-378 | *C. ciliaris* | 76.6 | 711.1 | 439.6 | 334.7 | 48.3 | 78.35 | 1.69 | 542.1 | 70.24 | 477.3 | 8.76 | 7.19 | 4.37 | 5.22 | 1.57 | 4.64 |
| IG-97-379 | *C. ciliaris* | 80.4 | 705.9 | 399.5 | 307.9 | 46.4 | 75.29 | 1.7 | 573.9 | 75.79 | 529.6 | 9.71 | 7.97 | 4.9 | 5.85 | 2.14 | 11.01 |
| IG-97-384 | *C. ciliaris* | 101 | 686.4 | 434.3 | 342.8 | 50.2 | 60.7 | 1.75 | 546.3 | 72.88 | 484.2 | 8.88 | 7.29 | 4.44 | 5.31 | 1.68 | 15.77 |
| IG-97-387 | *C. ciliaris* | 103.1 | 684.3 | 421.9 | 324.7 | 43.1 | 56.5 | 1.75 | 556.2 | 75.7 | 500.4 | 9.18 | 7.54 | 4.6 | 5.5 | 1.79 | 14.16 |
| IG-97-388 | *C. ciliaris* | 83.7 | 701.3 | 433.6 | 323.9 | 53.6 | 65.96 | 1.71 | 546.9 | 72.24 | 485.1 | 8.9 | 7.3 | 4.44 | 5.32 | 1.63 | 8.90 |
| IG-97-388-1 | *C. ciliaris* | 73.1 | 677.6 | 443.4 | 333.1 | 49.7 | 66.53 | 1.77 | 539.1 | 72.83 | 472.4 | 8.66 | 7.11 | 4.32 | 5.16 | 1.54 | 9.53 |
| IG-97-403 | *C. ciliaris* | 82.1 | 668 | 427.3 | 330.5 | 50.7 | 87.53 | 1.8 | 551.9 | 76.81 | 493.4 | 9.05 | 7.43 | 4.53 | 5.42 | 1.71 | 7.56 |
| IG-97-446 | *C. ciliaris* | 83.6 | 726.9 | 480.3 | 374.7 | 64.2 | 73 | 1.65 | 510.1 | 65.32 | 424.3 | 7.78 | 6.39 | 3.83 | 4.58 | 0.87 | 13.63 |
| EC400605 | *C. ciliaris* | 92.2 | 689.1 | 440.9 | 336.4 | 50 | 82.16 | 1.74 | 541.0 | 71 | 475.6 | 8.72 | 7.16 | 4.35 | 5.2 | 1.64 | 7.80 |
| IG-99-124 | *C. ciliaris* | 71.7 | 663 | 423.1 | 322.8 | 55.4 | 85.3 | 1.81 | 555.0 | 74.97 | 498.8 | 9.15 | 7.51 | 4.58 | 5.48 | 1.98 | 10.25 |
| IG-99-127 | *C. ciliaris* | 69.6 | 691.8 | 420.2 | 311.4 | 52.3 | 74.64 | 1.74 | 557.5 | 74.58 | 502.6 | 9.22 | 7.57 | 4.62 | 5.53 | 1.85 | 7.69 |
| IG-99-128 | *C. ciliaris* | 82.1 | 689.2 | 427.9 | 309.2 | 55.7 | 70.18 | 1.74 | 551.4 | 73.3 | 492.6 | 9.03 | 7.42 | 4.52 | 5.41 | 1.78 | 5.54 |
| IG-99-141 | *C. ciliaris* | 74.9 | 696.6 | 442.9 | 317.5 | 52.6 | 60.73 | 1.72 | 539.6 | 72.5 | 473 | 8.68 | 7.12 | 4.32 | 5.17 | 1.43 | 6.21 |
| IG-99-144 | *C. ciliaris* | 69.8 | 699.5 | 426.6 | 304.2 | 51.3 | 74.75 | 1.72 | 552.4 | 72.84 | 494.2 | 9.06 | 7.44 | 4.54 | 5.43 | 1.76 | 20.68 |
| IGFRI 727 | *C. ciliaris* | 85 | 696.4 | 431.5 | 331.8 | 50.5 | 56.53 | 1.72 | 548.5 | 72.34 | 487.9 | 8.95 | 7.35 | 4.47 | 5.35 | 1.71 | 11.13 |
| IGFRI S-3108 | *C. ciliaris* | 85.4 | 701.5 | 417.5 | 322.2 | 49.7 | 49.02 | 1.71 | 559.5 | 73.02 | 506.1 | 9.28 | 7.62 | 4.66 | 5.57 | 1.95 | 10.96 |
| EC397331 | *C. setigerus* | 94.3 | 671.3 | 420.1 | 315.3 | 46.1 | 48.87 | 1.79 | 557.4 | 74.45 | 502.8 | 9.22 | 7.57 | 4.62 | 5.53 | 2.02 | 8.74 |
| EC397393 | *C. setigerus* | 87.1 | 696.4 | 426.3 | 307.4 | 49.5 | 53.66 | 1.72 | 552.6 | 72.6 | 494.7 | 9.07 | 7.45 | 4.54 | 5.43 | 1.81 | 4.96 |
| EC397491 | *C. setigerus* | 88.8 | 727.7 | 427.7 | 318.6 | 53.3 | 49.91 | 1.65 | 551.4 | 68.29 | 492.8 | 9.04 | 7.42 | 4.52 | 5.41 | 1.87 | 5.75 |
| EC397557 | *C. setigerus* | 80.3 | 710.6 | 420.8 | 306.1 | 58.5 | 46.39 | 1.69 | 557.0 | 72.12 | 501.8 | 9.2 | 7.56 | 4.62 | 5.52 | 1.87 | 5.00 |
| IG96-82 | *C. setigerus* | 85.3 | 711.4 | 422.1 | 296.1 | 55.6 | 72.84 | 1.69 | 556.0 | 72.12 | 500.2 | 9.17 | 7.53 | 4.6 | 5.5 | 1.83 | 4.18 |
| IG97-425 | *C. setigerus* | 95.7 | 702 | 425.5 | 311.1 | 52.7 | 38.6 | 1.71 | 553.1 | 71.6 | 495.6 | 9.09 | 7.46 | 4.55 | 5.44 | 1.87 | 7.92 |
| IG97-426 | *C. setigerus* | 90.5 | 687.4 | 414.5 | 296.3 | 57.6 | 42.66 | 1.75 | 562.0 | 75.87 | 510.1 | 9.36 | 7.68 | 4.7 | 5.62 | 1.92 | 9.23 |
| IG97-454 | *C. setigerus* | 75 | 690.2 | 420.1 | 311 | 51.8 | 67.8 | 1.74 | 557.6 | 74.92 | 502.8 | 9.22 | 7.57 | 4.63 | 5.53 | 1.84 | 9.36 |
| IG97-455 | *C. setigerus* | 85.5 | 678 | 441.3 | 291.7 | 50.5 | 59.81 | 1.77 | 540.7 | 72.07 | 475.1 | 8.71 | 7.15 | 4.34 | 5.2 | 1.64 | 3.09 |
| IG97-457 | *C. setigerus* | 85.6 | 668.9 | 461.8 | 302 | 75.5 | 53.9 | 1.79 | 524.5 | 70.73 | 448.5 | 8.23 | 6.75 | 4.07 | 4.87 | 1.32 | 2.48 |
| IG97-464 | *C. setigerus* | 80.1 | 681.9 | 474.1 | 294.6 | 64.7 | 48.95 | 1.76 | 514.8 | 67.25 | 432.5 | 7.93 | 6.51 | 3.91 | 4.68 | 1.18 | 5.18 |
| EC400595 | *C. setigerus* | 89 | 714.5 | 428.8 | 321.1 | 55.1 | 40.91 | 1.68 | 550.7 | 71.67 | 491.4 | 9.01 | 7.4 | 4.51 | 5.39 | 1.69 | 10.23 |
| IG99-132 | *C. setigerus* | 78.7 | 699.7 | 430.3 | 323.6 | 54.6 | 66.47 | 1.72 | 549.3 | 71.2 | 489.4 | 8.98 | 7.37 | 4.49 | 5.37 | 1.8 | 10.12 |
| IG99-147 | *C. setigerus* | 92.7 | 703.3 | 421.4 | 333.7 | 56.1 | 49.42 | 1.71 | 556.4 | 72.28 | 501 | 9.19 | 7.54 | 4.61 | 5.51 | 1.9 | 12.27 |
| IG99-149 | *C. setigerus* | 80.4 | 707.1 | 441.8 | 312.8 | 56.6 | 61.83 | 1.7 | 540.3 | 69.05 | 474.5 | 8.7 | 7.15 | 4.34 | 5.19 | 1.63 | 7.99 |
| IG99-153 | *C. setigerus* | 102.7 | 651.7 | 390.1 | 285.3 | 59.9 | 68.11 | 1.84 | 581.2 | 82.16 | 541.8 | 9.94 | 8.16 | 5.02 | 6 | 2.35 | 11.83 |
| IG99-156 | *C. setigerus* | 85.7 | 656.3 | 414.3 | 321.4 | 45.6 | 33.59 | 1.83 | 561.8 | 74.93 | 510.3 | 9.36 | 7.68 | 4.7 | 5.62 | 2.24 | 6.88 |
| IG99-157 | *C. setigerus* | 88.7 | 674.3 | 400.8 | 295.1 | 65.6 | 59.99 | 1.78 | 572.8 | 78.36 | 527.8 | 9.68 | 7.95 | 4.88 | 5.83 | 2.17 | 11.41 |
| IG99-317 | *C. setigerus* | 76.8 | 692.8 | 413.7 | 315.7 | 57.1 | 46.61 | 1.73 | 562.6 | 74.97 | 511.1 | 9.37 | 7.7 | 4.71 | 5.63 | 1.96 | 7.64 |
| IG99-419 | *C. setigerus* | 82.2 | 715.9 | 432.8 | 314.2 | 62.9 | 66.5 | 1.68 | 547.4 | 68.75 | 486.3 | 8.92 | 7.32 | 4.46 | 5.33 | 1.8 | 3.33 |
| IG96-377 | *C. echinatus* | 73.2 | 740.2 | 451.1 | 318.9 | 69.9 | 37.92 | 1.62 | 532.9 | 64.55 | 462.3 | 8.48 | 6.96 | 4.21 | 5.04 | 1.52 | 6.08 |
| EC397342 | *C. echinatus* | 106.5 | 705.9 | 433.8 | 287.4 | 59.9 | 10.69 | 1.7 | 546.7 | 71.05 | 484.9 | 8.89 | 7.3 | 4.44 | 5.31 | 1.68 | 3.15 |
| EC397342-S1 | *C. echinatus* | 99.5 | 688.8 | 386.4 | 307.2 | 53.6 | 34.6 | 1.74 | 583.9 | 76.53 | 546.6 | 10.02 | 8.23 | 5.07 | 6.06 | 2.52 | 3.20 |
| EC397620 | *C. biflorus* | 104.6 | 689.4 | 446.3 | 341.2 | 55.8 | 58.34 | 1.74 | 536.7 | 69.58 | 468.6 | 8.6 | 7.06 | 4.28 | 5.12 | 1.62 | 7.80 |
| EC397325 | *C.*  *pennisetiformis* | 80.3 | 670.8 | 411.4 | 309.2 | 52.2 | 56.95 | 1.79 | 564.3 | 76.7 | 514 | 9.43 | 7.74 | 4.74 | 5.66 | 2.07 | 4.20 |
| EC397345 | *C.*  *myosuroides* | 75 | 665 | 388 | 303.5 | 37.8 | 62.04 | 1.8 | 582.9 | 80.79 | 544.5 | 9.99 | 8.2 | 5.05 | 6.03 | 2.38 | 1.85 |
| !CP=Crude protein g kg^-1^ DM; NDF = Neutral detergent fibre g kg^-1^ DM; ADF = Acid detergent fibre g kg^-1^ DM; Lignin= g kg^-1^ DM; Sugar = mg g^-1^ DM; DMI = Dry matter intake %; DDM = Digestible dry mater g kg^-1^ DM; RFV = Relative feed value %; TDN = Total digestible nutrients g kg^-1^ DM; DE = Digestible energy Mj kg^-1^ DM; ME =Metabolizable energy Mj kg^-1^ DM; NE_L_ = Net energy for lactation Mj kg^-1^ DM; NE_G_ = Net energy for growth/gain Mj kg^-1^ DM; NE_M_ = Net energy for maintenance Mj kg^-1^ DM; DMY=Dry matter yield t/ha | | | | | | | | | | | | | | | | | |
